# Neural Stem Cells and Cannabinoids in the Spotlight as Potential Therapy for Epilepsy

**DOI:** 10.3390/ijms21197309

**Published:** 2020-10-03

**Authors:** Diogo M. Lourenço, Leonor Ribeiro-Rodrigues, Ana M. Sebastião, Maria J. Diógenes, Sara Xapelli

**Affiliations:** 1 Instituto de Farmacologia e Neurociências, Faculdade de Medicina, Universidade de Lisboa, 1649-028 Lisboa, Portugal; diogo.lourenco@medicina.ulisboa.pt (D.M.L.); leonorrodrigues@medicina.ulisboa.pt (L.R.-R.); anaseb@medicina.ulisboa.pt (A.M.S.); diogenes@medicina.ulisboa.pt (M.J.D.); 2 Instituto de Medicina Molecular João Lobo Antunes, Faculdade de Medicina, Universidade de Lisboa, 1649-028 Lisboa, Portugal

**Keywords:** epilepsy, cannabinoids, neural stem cells, adult hippocampal neurogenesis

## Abstract

Epilepsy is one of the most common brain diseases worldwide, having a huge burden in society. The main hallmark of epilepsy is the occurrence of spontaneous recurrent seizures, having a tremendous impact on the lives of the patients and of their relatives. Currently, the therapeutic strategies are mostly based on the use of antiepileptic drugs, and because several types of epilepsies are of unknown origin, a high percentage of patients are resistant to the available pharmacotherapy, continuing to experience seizures overtime. Therefore, the search for new drugs and therapeutic targets is highly important. One key aspect to be targeted is the aberrant adult hippocampal neurogenesis (AHN) derived from Neural Stem Cells (NSCs). Indeed, targeting seizure-induced AHN may reduce recurrent seizures and shed some light on the mechanisms of disease. The endocannabinoid system is a known modulator of AHN, and due to the known endogenous antiepileptic properties, it is an interesting candidate for the generation of new antiepileptic drugs. However, further studies and clinical trials are required to investigate the putative mechanisms by which cannabinoids can be used to treat epilepsy. In this manuscript, we will review how cannabinoid-induced modulation of NSCs may promote neural plasticity and whether these drugs can be used as putative antiepileptic treatment.

## 1. Introduction

Epilepsy, one of the oldest known neurological diseases [1] was, for centuries, associated with a divine malady or demonic possession—and as such, exorcism was the only known therapy for this pathology [2]. Hippocrates in 400 B.C. demystified epilepsy by arguing that it was a medical problem that originated in the brain, instead of a problem of divine origin [1]. However, the prevailing supernatural view remained rather unchanged until the 17th century, when the first effective antiepileptic medicine, bromide, was introduced [3]. Antiepileptic drugs (AEDs), focal epilepsy surgery, vagus nerve stimulation, and the ketogenic diet are the available therapies to treat epilepsy. Nevertheless, the effectiveness of these therapies is highly affected by disease etiology (reviewed in Reference [4]).

Mounting evidence has suggested that adult hippocampal neurogenesis (AHN) is altered in patient and animal models of epilepsy [5,6], particularly during prolonged seizures, which acutely increase AHN, depleting the pool of neural stem cells (NSCs) [6,7,8]. However, the functional implications of this altered neurogenesis in epilepsy are still poorly understood.

Cannabinoids, used as a supplement or as an alternative to conventional AEDs, have been shown in multiple studies and reviews to have antiepileptic properties [9,10,11]. Enhanced by social networks and media coverage, these compounds have sparked intense interest among patients and the scientific community regarding the potential of medical-*Cannabis* to treat epilepsy.

Given the known role of cannabinoids in the regulation of AHN and their potential impact in the treatment of epilepsy [11,12], the present manuscript aims to review the mechanisms by which cannabinoids control epilepsy, specifically by looking at cannabinoid-induced modulation of NSCs.

## 2. Epilepsy and Epileptogenesis

Epilepsy is a common brain disease, affecting between 50 to 65 million people worldwide [13,14,15,16], and is characterized as a long-lasting propensity to engender epileptic seizures. These are defined by the International League Against Epilepsy (ILAE), as a transient occurrence of signs and/or symptoms, due to abnormal excessive or synchronous neuronal activity in the brain [17]. The ILAE also describes that patients with epilepsy, besides neurobiological problems, also face cognitive, psychological, and social issues [17].

Epileptic seizures can be classified depending on the onset as: Focal, if limited to one hemisphere; generalized, if rapidly spread bilaterally; or unknown, if the onset is unable to be determined, due to lack of information [18,19]. Under this big umbrella, seizures can also be categorized as motor or nonmotor, and they can be detailed depending on awareness (in case of focal seizures) [19]. In a broad view, motor behavior may include loss of tone (atonic), sustained stiffening (tonic), rhythmic jerks (clonic), irregular and brief jerks (myoclonic), flexion, or extension of arms, and flexion of trunk (epileptic spasms). Generalized motor seizures can comprise more than one motor behavior. Generalized non-motor seizures imply absence seizures. The type of epilepsy can also be classified as focal, generalized, combined generalized and focal, or unknown. Besides seizure and epilepsy type, diagnosis also comprises the recognition of epileptic syndromes (such as childhood absence epilepsy, Lennox-Gastaut syndrome, or Dravet syndrome) [18].

It is also important to know the etiology of epilepsy to adequate the best treatment. Epilepsy can be classified with different etiologies: Genetic, structural, infectious, metabolic, immune, unknown, or with more than one etiology [18]. Genetic etiology refers to seizures caused by a genetic mutation, such as in Dravet syndrome. Structural etiology implies the presence of acquired or abnormal genetic structures, such as in the hippocampus or amygdala, often associated with mesial Temporal Lobe Epilepsy (mTLE), which is the most common and studied form of epilepsy, and is frequently intractable. An infectious etiology is related to an infection, like cerebral malaria or the Zika virus, resulting in the appearance of seizures. A metabolic etiology is linked to metabolic disorders, such as porphyria. Finally, an immune origin is usually associated with central nervous system (CNS) inflammation mediated by autoimmune disorders (for example, autoimmune encephalitis) [18].

**Figure 1:**
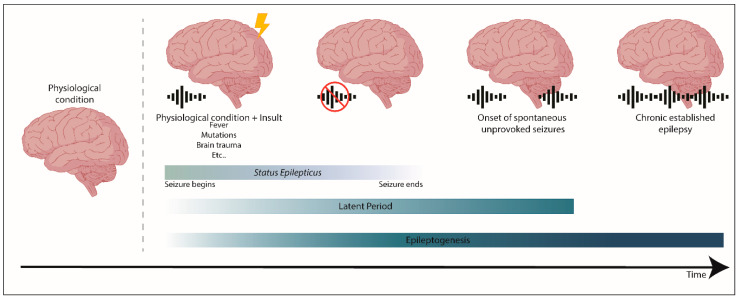
The process of epileptogenesis. Epileptogenesis encompasses three phases: (1) The moment a precipitating injury or event occurs, (2) the latent period, which comprises the time between an epileptogenic insult on a physiological brain, and the generation of a modified epileptic brain with spontaneous seizures, and (3) the mechanisms that occur during established chronic epilepsy. *Status epilepticus* (SE) is a prolonged seizure or a period of repetitive seizures without returning to the physiological state.

Epileptogenesis is when a physiological and functional brain develops recurrent and unprovoked seizures, due to abnormal biological alterations (reviewed in References [20,21]). Epileptogenesis encompasses: The moment a precipitating injury (such as stroke or traumatic brain injury) or event (as *status epilepticus* (SE) or febrile seizure) occurs; the latent period between this epileptogenic insult and a modified epileptic brain (having spontaneous unprovoked seizures); and the mechanisms that occur during chronic epilepsy (reviewed in References [20,21]) (Figure 1). It is worth mention that traditionally, the process of epileptogenesis was considered to stop at the time of the first spontaneous seizure (reviewed in Reference [22]).

Using mainly experimental models of mTLE it was possible to start unveiling the typical alterations that are involved in epileptogenesis [20,22,23]. However, the mechanisms underlying epileptogenesis are not yet fully understood. It is still not known which mechanisms are responsible for the generation of epilepsy or which are secondary or compensatory mechanisms that intend to repair the brain. The most frequent alterations observed in experimental mTLE models are neuronal cell death, mainly of hippocampal pyramidal cells [24], but also of hilar mossy cells, which are excitatory neurons in the hilus of the dentate gyrus [24], and of inhibitory GABAergic interneurons [25]; reactive gliosis [26]; blood–brain barrier damage [27]; alterations in the expression of GABA_A_ receptor subunit [28]; and changes in diverse signaling pathways, for example, brain-derived neurotrophic factor/tyrosine receptor kinase B (BDNF/TrkB), mammalian target of rapamycin (mTOR), or Janus kinase/signal transducers and activators of transcription (JAK/STAT) pathways [29] (reviewed in References [21,30,31,32,33]). Another important feature is the modifications in neurogenesis and associated-processes [24], which will be further detailed in this review. 

Despite that the knowledge about the epileptogenesis process has significantly increased, most of the current drugs for epilepsy are used to treat symptoms, meaning to stop the seizures. These drugs, named AEDs, do not prevent or cure epilepsy (reviewed in Reference [34]). Therefore, finding drugs that work as antiepileptogenic, interreacting with the process of epilepsy development, is fundamental. Current AEDs are mainly based on four mechanisms of action: (1) Modulation of voltage-gated ion channels (as valproic acid, phenytoin or carbamazepine); (2) enhancement of GABA-mediated inhibitory neurotransmission (like valproic acid, phenobarbital or tiagabine); (3) reduction of glutamate-mediated excitatory neurotransmission (as felbamate, perampanel or gabapentin) and (4) modulation of neurotransmitter release through presynaptic release machinery (as levetiracetam, gabapentin or pregabalin) (reviewed in References [32,34]).

Given the fact that about 30% of patients with epilepsy remain resistant to pharmacotherapy, continuing to experience seizures (reviewed in References [35,36]), it is imperative to persist studying the mechanisms underlying epileptogenesis. Developing innovative antiepileptogenic therapies that can modify this process, instead of only diminishing or abolishing seizures, and can decrease epilepsy-related comorbidities after the clinical diagnosis of epilepsy, is critical.

Experimental models, either in vivo or in vitro, mimic different types of epileptic seizures, syndromes, or specific aspects of the disease. In vivo animal models have been categorized into a different seizure or epilepsy models: Chemical or pharmacological (induced by pilocarpine, kainate or pentylenetetrazole (PTZ)); electrical stimulation (such as the kindling model or maximal electroshock seizures (MES)); genetic (mutations related to dysfunction of ion channels, receptors, enzymes or transporters); developmental (like the febrile seizures model) and trauma (as cortical undercut model) (reviewed in References [37,38,39]). These models are classified as models of epilepsy or models of seizures, depending on whether they result in chronic epilepsy or not, respectively [37].

The most commonly used models to mimic mTLE, are the pilocarpine (an acetylcholine receptor agonist), kainate (a glutamate analogue), or kindling models (a process that triggers epileptic seizures through repeated low-intensity electrical stimulation in a given brain region) (reviewed in References [38,40]). Pilocarpine- and kainate-induced SE models are more similar with the epileptic process occurring in humans than the kindling model, since they include an initial precipitating injury, a latent period, and finally, spontaneous, recurrent chronic seizures (reviewed in Reference [37]). On the other hand, the kindling model enhances seizure susceptibility, potentiating the generalization of electrical-induced seizures to other areas of the brain and ultimately, promoting spontaneous seizures (reviewed in Reference [38]).

In vitro models are mainly models of epileptogenesis, such as organotypic brain slices, or models of epileptiform activity, in which the biological preparations, like neuronal cultures or acute brain slices, are susceptible to chemoconvulsants, and therefore, epileptiform activity is acutely induced (reviewed in Reference [41]).

## 3. Adult Neurogenesis and Neural Stem/Progenitor Cells

NSCs are capable of self-renewal and give rise to cells of the neural lineage (neurons, astrocytes, and oligodendrocytes) (reviewed in Reference [42]). The process of generating functionally integrated neurons originated from NSCs is called neurogenesis, which includes the proliferation and differentiation of NSCs. In the adult mammalian brain, there are two well-known neurogenic niches: The Subventricular Zone (SVZ), lining the lateral wall of the lateral ventricles, and the Subgranular Zone (SGZ) of the hippocampal Dentate Gyrus (DG), a brain region highly affected in different types of epilepsy (reviewed in Reference [43]). The DG is formed by three layers, from the outer to the inner layer: Molecular layer (ML), granule cell layer (GCL), and hilus (also known as a polymorphic layer) [44,45].

**Figure 2:**
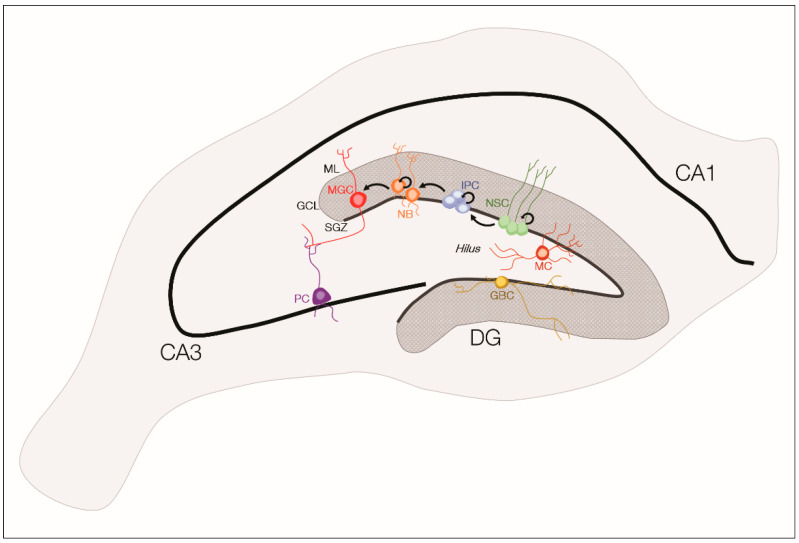
Schematic structure of the hippocampus and of adult hippocampal neurogenesis. The hippocampal dentate gyrus (DG) is formed by three layers—the molecular layer (ML), granule cell layer (GCL), and hilus. NSCs (or Type 1 cells) can be found in the Subgranular Zone (SGZ) and divide asymmetrically to generate intermediate progenitor cells (or Type 2 cells, IPC). These IPCs have proliferative capabilities and differentiate into neuroblasts (or Type 3 cells, NB) that will mature into granule cells (MGC), integrating the GCL and making functional connections with pyramidal cells (PC) of the CA3 region of the hippocampus. Inhibitory inputs from GABAergic basket cells (GBC) maintain NSCs in a quiescent state. Mossy cells (MC) are the predominant type of cells found in the DG hilus. CA1 = Cornu Ammonis 1; CA3 = Cornu Ammonis 3; DG = Dentate Gyrus.

The SGZ niche (Figure 2), located between the GCL and the hilus, contains NSCs (also known as Type 1 or radial glial-like precursor cells (RGLs)), which are maintained in a quiescent state, due to the inhibition from GABAergic basket cells [46]. When NSCs exit this state, they start proliferating and differentiating into Type 2 progenitor cells (also called intermediate progenitor cells, IPC) or into astrocytes (reviewed in Reference [47]). Unlike NSCs, which have a radial morphology with one apical dendrite projecting into the GCL and the ML, the Type 2 cells present few and short processes that are oriented tangentially to the length of the SGZ (reviewed in Reference [47]). Type 2 cells are known to receive GABAergic inputs from the hippocampal circuitry, which are excitatory and not inhibitory [48]. Moreover, Type 2 cells can be subdivided (into Type 2a or 2b) depending on the expression of certain markers and have a highly proliferative capacity, giving rise to Type 3 progenitor cells (also called neuroblasts) (reviewed in Reference [47]). These cells, with lower proliferative capacity, start migrating to the GCL where they slowly mature into immature neurons. These immature neurons in the postmitotic stage express different markers (as calretinin, and doublecortin (DCX), and integrate the GCL, extending their mossy fiber axons toward hippocampal CA3 pyramidal neurons and their dendrites toward the ML (reviewed in Reference [47]). At this stage, immature neurons receive only excitatory GABAergic inputs, which are important for maturation, synaptic integration, and dendritic development [48,49]. Later, when neurons start forming synapses with cells from CA3 and ML, GABAergic inputs begin to exert an inhibitory action instead of excitatory and glutamatergic inputs start to emerge [48,49].

After these developmental processes, NSC-derived immature GABAergic neurons culminate maturing into glutamatergic neurons expressing neuronal nuclei (NeuN) and calbindin. It is relevant to emphasize that just a small fraction of new neurons survive and integrate hippocampal circuitry and the remaining cells suffer apoptosis before establishing connections [50,51]. Importantly, the process of generation and maturation of new neurons has been implicated in hippocampus-dependent spatial learning, formation, and integration of new memories, pattern separation, anxiety- and depressive-related behaviors (reviewed in References [43,52,53]).

### 3.1. Neural Stem Cells in Epilepsy

Given that the hippocampus is affected in different types of epilepsy, studying AHN in the context of epilepsy is vital. In fact, the tight regulation of immature and mature neurons by GABAergic cells can be disrupted by epileptic seizures (reviewed in Reference [54]). Data obtained from human patients and rodent models of mTLE, have shown that convulsive seizures induce several molecular and morphological abnormalities leading to aberrant neurogenesis [55,56,57]. Interestingly, although these alterations are more extensive when promoted by convulsive seizures, non-convulsive seizures may also induce these abnormalities [58].

Epileptic seizures promote an initial increase of AHN by inducing an excessive activation of NSCs in kainate-induced SE or epileptiform activity model [59] and cell proliferation in the DG in pilocarpine-induced SE or kindling model [55,60]. However, in the presence of an epileptic trigger, like kainate, NSC fate is modified, differentiating mainly into reactive astrocytes, costing the maintenance of the NSC pool [59]. Therefore, in the long term, the NSC pool is depleted by seizures, and a permanent decline of neurogenesis is observed in this context [59]. Furthermore, in pharmacological and kindling animal models, newly seizure-generated hippocampal neurons have altered morphological properties with an abnormal generation of basal dendrites extended toward the hilus, called hilar basal dendrites, which make anomalous synapses with mossy fiber axons [55,61,62]. The mossy fiber axons from immature and mature neurons can also start sprouting aberrantly, targeting the apical dendrites of granule cells in the ML, thus, promoting non-physiological connections, as observed in the pilocarpine-induced SE model [55,63].

Another striking seizure-induced alteration is the incorrect migration and integration of differentiating cells in the GCL. In fact, seizures induce the appearance of ectopic granule cells in the hilus and the consequent dispersion of the GCL, either in the pilocarpine-induced SE model or in human patients with mTLE (reviewed in Reference [55]). Pathologic proliferation of neuroblasts or the loss of reelin, a protein involved in neuronal migration, may be on the basis of the atypical migration in pharmacological models of epilepsy [64,65]. Albeit these ectopic granule cells receive typical inputs from the perforant path [66], they also fired synchronously with CA3 pyramidal cells, which may contribute to the generation of spontaneous seizures [67] in pharmacological models of epilepsy.

The above-described abnormal neurogenesis, from the incorrect differentiation of NSCs to the development of atypical neuronal processes and their migration, originating hilar-ectopic granule cells, dramatically disturbs physiological synaptic transmission within the hippocampal network. The development of new synapses of mossy fiber axons with basal dendrites in the inner layer of the DG and the synchronization of misplaced granule cells with CA3 pyramidal cells lead to a recurrent excitatory loop, most probably causing hippocampal-dependent memory impairments [68,69,70].

It is not completely clear whether aberrant neurogenesis is a contributing factor for epilepsy or the cognitive deficits, which often manifests in patients with epilepsy, or if it is a compensatory mechanism of the brain in an attempt to self-repair. Some reports demonstrate that seizure-generated granule cells exhibit reduced excitability, while inhibition of AHN did not affect or enhance excitability in the kindling model [71,72,73]. However, the theory that AHN blockade may reduce seizures and cognitive impairment is gaining strong evidence and is starting to be widely accepted [68,74,75,76]. In 2004, an antimitotic agent infused in epileptic rats decreased the development of spontaneous recurrent seizures and reduced the number of ectopic granule cells in the hilus and astrocytes in CA1, although mossy fiber sprouting was not shown to be altered [74]. These same positive effects were also demonstrated in the presence of the anti-inflammatory Celecoxib, a selective cyclooxygenase-2 (COX-2) inhibitor, in the pilocarpine-induced SE model [75]. Concomitantly, Celecoxib inhibited NSC proliferation, prevented neuronal death and microglia activation [75,77]. Later, in 2007, Jessberger and colleagues showed that a widely used antiepileptic, valproic acid, normalized the seizure-induced cell proliferation and generation of new neurons, while decreasing the formation of hilar basal dendrites, thus, blocking aberrant neurogenesis in epileptic rats [68]. Moreover, valproic acid prevented the hippocampus-dependent memory impairment associated with seizures [68]. Furthermore, in 2015, the genetic ablation of adult-generated granule neurons was shown to reduce spontaneous recurrent seizures and prevent aberrant neurogenesis-related processes, such as cell proliferation and ectopic granular cell formation, in addition to preserving hippocampus-dependent memory [76].

One aspect that needs further research is the impact of the administration of AEDs on embryonic and postnatal neurogenesis. In recent years, studies done in vitro and in vivo with rodent models of epilepsy and in humans have highlighted some of the consequences [78,79,80]. A study performed in pregnant mice treated with valproic acid has shown that, in the offspring, embryonic neurogenesis is untimely upregulated, leading to an increase in cell proliferation, which in the adult, will consequently deplete the NSC pool and decrease the levels of adult neurogenesis in the hippocampus. This study also reported abnormal morphology and activity of hippocampal neurons [78]. In vitro, rat NSCs treated with ethosuximide, an AED used for absence epilepsy, were shown to have increased cell proliferation and neuronal differentiation [79]. Importantly, embryonic exposure to AEDs has worse outcomes than exposure in adulthood, with most animals presenting impaired spatial memory and cognitive deficits, resulting from neuronal death and structural changes in the rodent brain (reviewed in References [81,82]). However, translating this knowledge into humans has several limitations. Nevertheless, using magnetic resonance imaging (MRI), Ikonomidou and colleagues managed to indirectly measure neurogenesis by finding structural changes and significant decreases of grey matter volumes in the brains of adults that, in utero, were exposed to AEDs. These structural changes also resulted in a significant decrease in the IQ of adults that were prenatally exposed to AEDs when compared to control subjects [80]. A common conclusion from these experiments is that some AEDs impact embryonic and adult neurogenesis, whether by increasing or decreasing NSC proliferation or by delaying maturation and differentiation of neurons, as well as by influencing different processes in the context of brain morphogenesis and network formation [81,83].

### 3.2. Neural Stem Cell Therapies for Epilepsy

Seizure-induced neurogenesis may be a potential therapeutic target to attenuate the development of epilepsy and to prevent or rescue cognitive impairment in epilepsy. Given its importance in physiological conditions, AHN should not be completed inhibited. Therefore, strategies to prevent or block aberrant neurogenesis must be developed, to maintain the beneficial effects of newly generated neurons in cognitive functions. Many studies have great pre-clinical value showing the contribution of aberrant neurogenesis to epilepsy and cognitive impairment. However, the strategies applied are still not yet translatable to humans [84]. In this review, we focus on potential therapeutic approaches using NSCs in epilepsy that may be translated into clinical practice in the future (Table 1).

An approach using microRNAs (miRNAs) has been developed to modulate a few key aspects of aberrant neurogenesis and decrease seizures. miR-22 inhibition in a mouse model of epilepsy exacerbated the ectopic migration of new-born neurons [85] and promoted epileptic seizures, while impairing cognitive performance [86]. However, this cognitive impairment, tested through the novel object re-location test, could be related to the fact that epileptic mice injected with the miR-22 inhibitor (antagomir-22) were more anxious. Consistent with miR-22 suppressing aberrant neurogenesis, mice injected with this miRNA also demonstrated an anti-seizure effect [86]. Silencing miR-134 reduced CA3 pyramidal neuronal death and dendrite spine density and diminished aberrant mossy fiber sprouting [87,88]. Albeit the observed decrease in seizure severity, the impact of miR-134 on cognition was not tested [87,88] or was not changed [89]. Also, miR-135a controls hippocampal neuronal morphology and synaptic function [90]. Antagonizing this particular miRNA reduced spontaneous recurrent seizures; nevertheless, the impact in memory and learning processes is still not known [90]. Conversely, miR-135a inhibition in healthy mice stimulated neural precursor cell proliferation and differentiation into neurons without promoting astrogliogenesis [91]. Also, its inhibition was sufficient to restore the age-associated reduction of adult neurogenesis [91]. However, whether the potentiation of neurogenesis reduced by epilepsy would be beneficial and improve cognitive functions has yet to be revealed. Using miRNAs as a therapeutic approach has gained value overtime, and in fact, an RNA-based therapy has already been approved by the FDA as a treatment for polyneuropathy [92].

Another strategy that can be applied as a potential therapy for epilepsy is grafting medial ganglionic eminence (MGE) cells in the hippocampus (reviewed in References [93,94]). These cells are present in embryonic and fetal stages and differentiate into GABAergic interneurons. Given the evidence that seizure-induced neuronal loss is mainly from inhibitory GABAergic interneurons, this cell therapy would overcome this hallmark of epilepsy and help reducing seizures. In fact, multiple studies have already shown that MGE-grafts originate GABAergic interneurons, which are incorporated in the hippocampal network, prompting inhibitory synaptic neurotransmission by decreasing recurrent spontaneous seizures [95]. MGE cells derived by either human embryonic/fetal stem cells or from human-induced pluripotent stem cells (hiPSCs) appears to alleviate spontaneous seizures [95,96]. Nonetheless, there are ethical issues to consider when using embryonic/fetal stem cells in clinical trials (reviewed in Reference [93]). Using hiPSCs-derived cells also raises some concerns, mainly due to their instability and possibility to suffer epigenetic alterations or their high proliferative rate (reviewed in Reference [93]).

In a recent study, grafting of hiPSCs-derived MGE cells in the hippocampus of epileptic rats has shown that it not only reduced seizures, but also decreased mossy fibers sprouting and ectopic hilar granule cells generation, which preserves reelin-positive neurons and other interneurons, and increases the number of newly born neurons in the SGZ-GCL [96]. Moreover, this approach alleviated cognitive and memory impairments and mood dysfunction, another comorbidity associated with epilepsy [96]. It is worth noting that cognitive impairment recovery was also achieved with human embryonic stem cells [95]. Although safety concerns are associated with a grafting approach, clinical translation has already started, as clinical trials using hiPSC have been approved to treat age-related macular degeneration or Parkinson’s Disease [97,98]. The great advantage of using hiPSC is the fact that they derive from the patient’s somatic cells and are autologously transplanted after reprogramming [94]. Thus, it makes sense to continue to put efforts in cell therapy research to understand its impact on modulating neurogenesis and enhance cognition, besides decreasing seizures.

Both the miRNAs approach and the MGE cell therapy could reduce the number of cases with refractory epilepsy. Although these therapies may function as antiepileptic agents by modulating stages of neurogenesis and diminishing seizures, their impact on cognition impairment requires more attention, and their long-term effect also needs further studies.

A potentially less invasive therapy has also been studied for the treatment of epilepsy (reviewed in References [99,100]). Extracellular vesicles (EVs) derived from mesenchymal stem cells (MSC) and delivered intranasally in mice can revert the epileptogenic processes [101]. EVs, specifically A1 exosomes, secreted by human bone marrow-derived MSC were administered intranasally twice over 24h after pilocarpine-induced SE termination. These EVs reached the hippocampus within 6h and exerted neuroprotective and anti-inflammatory actions. After SE, delivered EVs diminished the loss of neurons and interneurons in the DG and the CA1 regions and recovered neurogenesis. Animals that received EVs after SE showed, six weeks later, a rescue of the number of immature neurons and dentate hilar neurons positive for reelin and decreased the number of ectopic new-born neurons, when compared to animals without EVs after SE. In addition, EVs were able to prevent SE-induced impairment in memory, cognition, and pattern separation. Furthermore, the administration of these vesicles reduced both microglia activation and proinflammatory cytokines levels, while increasing anti-inflammatory cytokines. Albeit the great impact of these vesicles on neurogenesis, cognition, and inflammation, thus, reversing the epileptogenic processes, the actual effect on seizures is not known. EVs were not administered during SE, so the ability of this approach to ameliorate spontaneous seizures remains to be determined. However, these EVs may work as an adjuvant therapy to AEDs, thus, having a synergistic antiepileptogenic and antiepileptic action. In addition, further studies are needed to confirm these abovementioned evidence and to evaluate the impact of EVs on mossy fiber sprouting and basal dendrites formation and to assess the long-term effects of this approach [101].

## 4. Cannabinoids and the Endocannabinoid System

*Cannabis* is a genus of plants indigenous to Central Asia where three species have been phylogenetic identified: *Cannabis sativa*, *Cannabis indica*, and *Cannabis ruderalis*. These plants have been widely used throughout History, due to its broad variety of applications, ranging from therapeutic and recreational use, religious purposes, to produce food for livestock, and for its fibers, to manufacture clothing [102,103]. According to the World Drug Report 2019, *Cannabis*-derived products (dried leaves, flowers, stems, seeds, and oils) are consumed by 3.8% of the world population (≈188 million people), a trend that, despite having increased in the past decade, has remained stable in the last years. This makes *Cannabis* one of the most consumed drugs, after alcohol, tobacco, or caffeine [104].

More than 560 phytocannabinoids (naturally occurring cannabinoids) have now been identified as constituents of the *Cannabis* plant. The most abundant cannabinoid present in the *Cannabis* plant is delta-9-tetrahydrocannabinol (Δ^9^-THC) [105]. The psychotomimetic effects of cannabinoid consumption include euphoria, appetite stimulation, sedation, altered perception, impairments in motor control, and memory deficits [106]. These effects are almost exclusively related to Δ^9^-THC, which was first isolated in its pure form and structurally described in 1964 by Mechoulam and colleagues [107]. Regardless of its psychotomimetic effects, Δ^9^-THC has therapeutic value and unique applications [108].

A growing body of scientific data has been attesting the immense potential of these cannabinoids to ameliorate symptoms of several pathologies. Indeed, medical-*Cannabis* is being used or proposed to treat glaucoma, depression, neuralgia, neurodevelopmental forms of refractory epilepsy, and cancer [103,109,110,111,112,113,114,115,116,117]. Particularly, *Cannabis* usage has been helping patients suffering from multiple sclerosis-associated neuropathic pain and tremors, ameliorating tremors and bradykinesia in Parkinson’s disease patients, relieving neuropsychiatric symptoms shown by most individuals with Alzheimer’s disease and in anxiety/mood disorders [118,119,120,121,122,123].

Moreover, Δ^9^-THC has been used to alleviate symptoms associated with several conditions, such as muscular spasticity, eating disorders, nausea, and vomiting after chemotherapy treatments, and weight-loss related to Acquired Immunodeficiency Syndrome (AIDS) [103,124,125,126,127,128]. Besides Δ^9^-THC, there are also non-psychotomimetic cannabinoids with several therapeutic functions, such as cannabichromene (CBC), cannabidiol (CBD), cannabidivarin (CBDV), cannabigerol (CBG), cannabinol (CBN), cannabivarin (CBV), delta-8-tetrahydrocannabinol (Δ^8^-THC), and delta-9-tetrahydrocannabivarin (Δ^9^-THCV) [105,129].

Notwithstanding, the chronic consumption of *Cannabis* has been correlated with detrimental health effects. Heavy and prolonged *Cannabis* use is associated with cognitive and memory impairments, increased probability of developing schizophrenia-spectrum disorders, acute psychosis, and mania [130,131,132,133,134]. When inhaled, *Cannabis* abuse can result in chronic bronchitis and impaired respiratory function [103,135]. Therefore, one of the challenges of *Cannabis* research is to find ways to prevent negative side-effects associated with *Cannabis*-based medicines [103].

### 4.1. The Endocannabinoid System in Physiological Conditions

The Endocannabinoid System (ECS) is a biological phylogenetic conserved system that can be found in both vertebrate and invertebrate organisms [136,137]. The ECS is comprised of the endocannabinoids (eCB), which are endogenous lipid-based neurotransmitters, their synthetizing and degrading enzymes, the cannabinoid receptors 1 and 2 (CB1R and CB2R), the endocannabinoid membrane transporter (EMT), and the CB1R interacting protein 1a [103]. Importantly, despite being considered part of the ECS, a putative EMT has yet to be identified (reviewed in References [138,139]).

**Figure 3:**
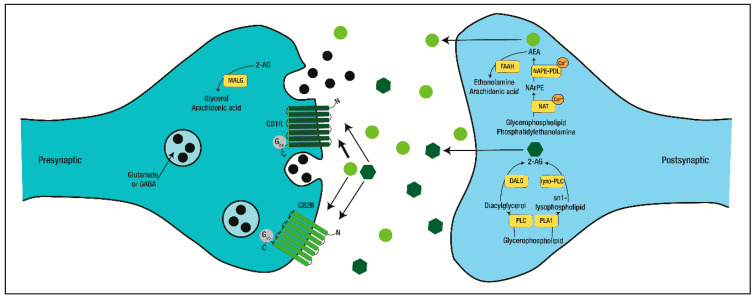
Pharmacology of Endocannabinoids. Anandamide (AEA) and 2-arachidonoglycerol (2-AG) are lipid-based neurotransmitters mainly synthesized postsynaptically and released “on demand” to the synaptic cleft, where they bind to the cannabinoid receptors (CB1R and CB2R) to modulate synaptic transmission.

The two best known, characterized, and studied eCBs are the N-arachidonoylethanolamine (anandamide, AEA) and 2-arachidonoglycerol (2-AG) [103], being AEA less abundant than 2-AG [140,141]. eCBs are synthesized “on demand” mainly from phospholipid precursors in cell membranes, and unlike classical neurotransmitters, are not stored in vesicles [103]. In fact, membrane glycerophospholipids can be cleaved by phospholipase C (PLC), forming diacylglycerol, which by action of diacylglycerol lipase (DAGL), synthesizes 2-AG. Alternatively, phospholipase A1 (PLA1), releases an sn-1 lysophospholipid from the membrane, which is cleaved by lyso-PLC to generate 2-AG [140]. AEA, on the other hand, is synthesized when calcium-dependent trans-acylases (NAT) act on glycerophospholipids and phosphatidylethanolamine, forming an N-arachidonoyl-phosphatidyl ethanolamine (NarPE) which is cleaved by calcium-dependent NAPE (N-acyl-phosphatidylethanolamine)-specific phospholipases D (NAPE-PLD) [140]. Hydrolysis of 2-AG and AEA occurs pre- and postsynaptically, respectively (Figure 3).

2-AG is hydrolyzed by monoacylglycerol lipase (MAGL) into arachidonic acid and glycerol. AEA is hydrolyzed via fatty acid amide hydrolase (FAAH) into arachidonic acid and ethanolamine [140]. When released to the synaptic cleft, eCBs act mainly retrogradely modulating presynaptic glutamatergic or GABAergic signaling by binding to CB1R or CB2R [103]. Additionally, AEA and 2-AG have different affinities for the cannabinoid receptors, with AEA having more affinity to CB1R than to CB2R, while 2-AG displays the same level of affinity for both receptors [103]. AEA works as a partial agonist for CB1R, whereas 2-AG binds with low affinity but works as a full agonist [142,143].

eCB release can take place either phasically (in an activity-dependent manner), or tonically (under basal conditions) [144,145]. Interestingly, there are also reports suggesting that eCB signaling can also occur in a non-retrograde mode, via postsynaptically located CB1Rs or via autocrine signaling, by direct activation of transient receptor potential vanilloid receptor type 1 (TRPV1), which belongs to the endovanilloid system, in which AEA is known to act as a full agonist [146,147,148].

Throughout the central and peripheral nervous system (PNS), eCBs modulate a broad range of physiological functions, such as the regulation of intestinal motility, fertility, circulatory system, myocardial function, myogenic, adipogenic, and osteo- and chondrogenic differentiations, metabolism, and inflammation [149,150,151,152,153,154,155]. These actions are mediated by the activation of the G protein-coupled domain receptors (GPCR) CB1R and CB2R [156].

Originally, these receptors were thought to be exclusively associated with G_i_ or G_o_ dependent inhibition of adenylyl cyclase activity. However, recent data has pointed out that coupling with both G_s_ and G_q_ can occur for CB1R, but not for CB2R [156,157,158]. Cannabinoid receptors activation has also been linked to the modulation of synaptic activity by transiently suppressing transmitter release (short-term depression, STD) or persistently (long-term depression, LTD) [145]. Importantly, eCBs modulate both inhibitory (depolarization-induced suppression of inhibition, DSI) and excitatory synaptic transmission (depolarization-induced suppression of excitation, DSE) [159,160]. CB1R and CB2R have also been linked to neuroprotection by controlling excessive excitatory transmission and calcium release, thus, preventing excitotoxity [161].

In summary, cannabinoid receptors can be considered to have few “intrinsic” signaling properties, and the physiological effects mediated by their activations are largely dependent on cell type, location, functional state, and temporal constraints (reviewed in References [157,162]). All these variables can affect the action of endo, phyto, and synthetic cannabinoids.

### 4.2. Cannabinoid Pharmacology and Actions in Physiological Conditions

**Figure 4:**
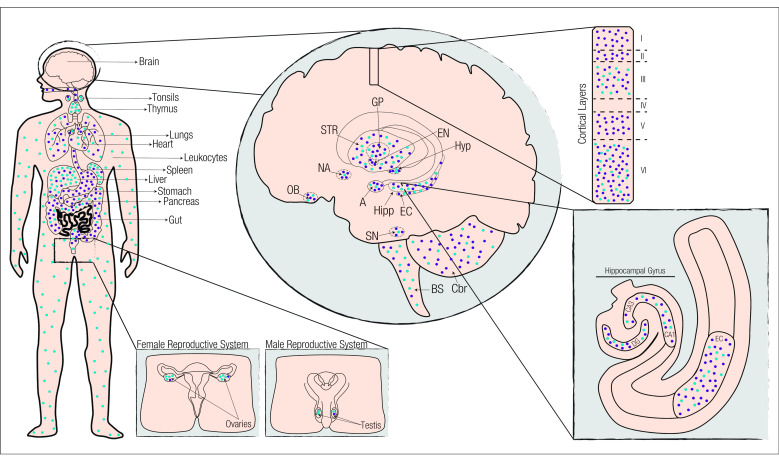
Expression of cannabinoid receptors in the CNS and peripherical tissues. The cannabinoid receptors display a differential expression pattern dependent on the tissue. CB1R (dark blue) has higher expression on the CNS than in the periphery. CB2R (light blue), although predominantly expressed in the periphery is also expressed in the CNS. A = Amygdala; BS = Brainstem; CA1 = Cornu Ammonis 1; CA3 = Cornu Ammonis 3; Cbr = Cerebellum; DG = Dentate Gyrus; EC = Entorhinal córtex; EN = Entopeduncular nucleus; GP = Globus Pallidus; Hipp = Hippocampus; Hyp = Hypothalamus; NA = Nucleus accumbens; OB = Olfactory Bulb; SN = Substantia Nigra; STR = Striatum.

Immunohistochemical and mRNA analysis of CB1R and CB2R, identified CB1R as the most abundant cannabinoid receptor in the CNS, and although CB2R has higher expression in peripheral tissues, such as the immune and digestive systems, new in situ hybridization and RT-PCR techniques identified CB2R in the CNS (Figure 4 and Supplementary Table S1).

Excitingly, CB2R levels can be enhanced in microglia and astrocytes following specific insults (such as neuroinflammation), and in certain conditions, like stroke. Importantly, the lack of psychotomimetic effect associated with CB2R modulation, is slowly attracting this receptor as a promising therapeutic target [163,164,165,166].

The abundance of cannabinoid receptors in different areas of the brain is a very strong indicator of the importance of the ECS on a variety of physiological functions [137,167]. Δ^9^-THC, the “classical cannabinoid”, exhibits a very distinct pattern of symptoms, known as the Tetrad Symptoms—hypothermia, analgesia, hypoactivity, and catalepsy. These have been the basis and have set the standard of cannabinoid research in a clinical setting [168,169]. Δ^9^-THC presents a mixed agonist-antagonist profile when binding to CB1R and CB2R varying according to cell type, concentration, receptor expression, and presence of other endo- and exo-cannabinoids acting as full agonists [170,171]. Because of the higher expression of CB1R (compared to CB2R) in the CNS, this receptor is currently thought to be responsible for the psychotomimetic effects of exogenous cannabinoids. This is supported by the fact that, by using selective CB1R antagonists, the cannabinoid-induced tetrad effects of cannabinoids can be effectively abolished [116,172]. Interestingly, Δ^9^-THC can also bind to other receptors, such as GPR55, serotonin, opioid, glycine, and peroxisome proliferator-activated receptor gamma (PPARγ) receptors. All of these receptors may also account for some of the effects described for this phytocannabinoid [105]. Conversely, evidence shows that while Δ^9^-THC has been shown to have no effect for TRPV1, it acts on another transient receptor potential (TRP) channels, such as TRPV2 and TRPA1 [105,173].

CBD is another cannabinoid very well described and studied, being devoid of psychotomimetic effects. These effects have been shown to result from its anti-inflammatory, antioxidant, antiepileptic, antirheumatic, anxiolytic, and analgesic properties, having therefore high medical value [174]. When used in conjunction with Δ^9^-THC, CBD positively modifies Δ^9^-THC induced-psychoactivity, increasing its clinical efficacy and duration of beneficial effects, such as reducting congestion, nausea, and promoting neuroprotection [108,175]. Ligand-binding assays have shown that CBD has a low affinity for both cannabinoid receptors, exhibiting agonistic activity for TRPV1 and for GPR55 [176,177,178]. It has been suggested that CBD can act as a non-competitive antagonist or even as an inverse agonist [179]. This behavior is heavily influenced by the dose of CBD administered, which may account for the variety of actions modulated in different tissues [179] (reviewed in Reference [180]). Therefore, CB1R and CB2R pharmacological action of CBD is still controversial, and further research is needed to reach scientific consensus.

Some authors have suggested that cannabinoids exert an “entourage effect” on the organism, meaning that besides Δ^9^-THC, other cannabinoids present in the *Cannabis* plant may act synergistically to modulate the systemic psychotomimetic effects of the plant [181]. This effect, however promising it may be, has nonetheless been questioned. Recently, Cogan [182], showed that clinical data suggest a lack of support of the “entourage effect” as a reliable phenomenon that is predictive of beneficial outcomes of *Cannabis* exposure.

### 4.3. The Endocannabinoid System in Epilepsy

Evidence from multiple animal models of epilepsy has shown that the ECS is dynamically altered following acute and chronic seizures [183]. In both kainate and pilocarpine models of induced-SE, the endogenous levels of AEA and 2-AG are increased [184,185,186,187], suggesting that an on-demand release of eCBs may trigger a neuroprotective action against seizure-induced neurotoxicity.

Cannabinoid receptors have been shown to be involved in the severity of induced seizures. Studies with CB1R conditional mutant mice (lack expression of the CB1R in principal forebrain neurons but not in GABAergic interneurons) have been used to study excitotoxicity and the involvement of CB1R in synaptic activity in epilepsy. When injected with kainate, these mutant mice displayed an increase in both gliosis and apoptosis and displayed excessive seizures in vivo [188]. Additionally, using this conditional CB1R knockout mice in the kindling paradigm, mice presented a lower seizure susceptibility and, a pharmacological blockade of TRPV1 had no effect on the duration of behavioral or electrophysiological seizure activity, showing the involvement of the ECS rather than of the endovanilloid system. Conversely, deletion of CB1R from GABAergic forebrain interneurons had no impact on either initial after discharge thresholds or post-kindling thresholds, again with no apparent involvement of the endovanilloid system [189].

Taken together, these findings suggest that CB1R expression is an important regulator of seizure duration, which is dependent on neuronal subpopulation expression. On the other hand, overexpression of CB1R in pyramidal neurons of the hippocampus promoted a reduction of induced-seizure severity and cell death [190].

After an acute seizure event, CB1R expression levels have been shown to be altered in both humans and in the pilocarpine mouse model of epilepsy. In patients, CB1R is shown to be downregulated in glutamatergic synapses [191]. On the other hand, mice subjected to the pilocarpine-induced SE show different expression levels based on whether they were classified as “weak” or “strong” animals. This classification is based on behavioral signs that, according to the modified Racine scale [192,193], separates animals according to their seizure activity. “Weak” animals, which develop mild seizures, showed a control-like phenotype, with no major changes in the distribution and density of CB1R in the hippocampus [194,195]. Contrastingly, “strong” animals (where seizures show intense motor symptoms), present a decrease in the expression of CB1R in the hippocampus [195].

In chronic epilepsy, when hippocampal sclerosis is present in patients (meaning shrinking and atrophy of hippocampal CA1 and CA3 regions, with increased cell death in CA1 area), data regarding CB1R expression is contradictory, with data suggesting both an increase [194] or a decrease [191] of CB1R expression in the hippocampal region. Interestingly, data that suggests an increase in the expression CB1R agrees with data from animal models in which, in the chronic stage of epilepsy (1–2 months after pilocarpine model induction), “strong” animals show an increase in the levels of CB1R throughout the hippocampus when compared to control mice [194,195].

Additionally, data from patients with epilepsy suggested that an increase in CB1R was negatively correlated with a latency in the frequency of seizures. Thus, it has been proposed that CB1R modulation may prove to be a disease modifier acting as antiepileptogenic by providing a protective mechanism for neurons against hyperexcitability and seizure activity, or contributing to the overall process of epileptogenesis, or both [196].

These dynamic changes in the expression of CB1R limit neuronal network disinhibition, allowing elevated neuronal excitability during prolonged epileptiform events [194]. Taken together, the observed changes in CB1Rs and eCBs in epilepsy, in both acute and chronic events, suggest that they may be related to the etiology of seizures or associated with developmental problems [197,198], hinting to a putative primary impairment of the ECS in epilepsy which may promote pharmacoresistant epilepsy.

More recently, CB2R has also been shown to be systemically activated to modulate immune-mediated symptoms associated with seizure-induced-neuroinflammation [199,200] (reviewed in References [201,202]). Interestingly, CB2R knockout mice exhibited an increased susceptibility to induced seizures [203]. Likewise, in the pilocarpine rat model, CB2R expression was upregulated in the hippocampus following SE [204]. Taken together, both these studies suggest a putative neuroprotective action of eCBs via CB2R in epilepsy [203,204].

Besides the abovementioned changes in the cannabinoid receptors, the production of eCBs in patients with epilepsy is also affected, as the levels of AEA, and of the cannabinoid synthesizing enzymes DAGL and MAGL are reduced, suggesting a pivotal role of cannabinoid tone in this disease [191,205].

Neuronal hyperexcitability in the hippocampus during SE has also been linked to the activation of TRPV1, which was also shown to be overexpressed [206]. In fact, recent data for both human patients with mTLE and animal models of epilepsy supported an increased TRPV1 expression in the temporal cortex and hippocampus [207,208,209]. Interestingly, TRPV1 expression was found to co-localize with CB1R in post-synaptic neurons in the hippocampus [210]. Capsazepine, a TRPV1 antagonist, when co-administered with CB1R/CB2R agonist WIN 55,212-2, managed to potentiate WIN antiepileptic effects, suggesting an interplay between the endocannabinoid and vanilloid systems in the regulation of hyperexcitability [208].

### 4.4. Cannabinoids and Neural Stem Cells

Studies taking advantage of transgenic animals for the cannabinoid receptors and their pharmacological manipulation with agonists and antagonists have put in evidence that the ECS modulates both embryonic and postnatal neurogenesis [12,211,212,213,214,215]. This system acts upon neural progenitor proliferation, differentiation, and the survival of adult NSCs, commitment, and maturation. It regulates the migration of cortical neurons and interneurons, and is a key player in axon guidance, pathfinding, and synaptogenesis [216,217,218,219,220,221].

Embryonically, eCBs have a crucial role in the creation of correct brain architecture and circuit wiring [222]. In fact, perinatally, eCB signaling, through CB1R and CB2R, promotes cell proliferation in the embryonic ventricular and subventricular zones [223]. Interestingly, cannabinoid receptor expression is quite low on neuronal progenitors and gradually increases with a neural commitment [217,224]. Radial migration of postmitotic neurons and tangential migration of immature interneurons to their final position in the cortical plate is also tightly regulated by eCBs [225,226]. Type-specific neuronal identification, synaptic button sprouting, and axonal migration are CB1R dependent [227]. All of these mechanisms can be abruptly disturbed and altered by phytocannabinoids, as these can pass the placental barrier [228]. In this critical developmental period, changes in eCB signaling can have an impact on cognition and behavior in the adult (reviewed in References [229,230]). In fact, maternal use of *Cannabis* during pregnancy induced an increase in susceptibility of neonatal problems, namely, preterm birth, pre-eclampsia, and neonatal intensive care [231,232], while later children display cognitive deficits, namely, in executive function, working memory tasks, sustained attention and learning, as well as psychiatric disorders [233,234] (reviewed in Reference [228]).

Postnatally, and similar to the effects seen embryonically, endo- and exogenous cannabinoids act on NSCs in the SVZ and SGZ niches. Studies using CB1R or CB2R-KO animals have put in evidence a regulatory role of these receptors in adult neurogenesis [214,235]. Pharmacological action using CB1R and CB2R agonists suggested that activation of these receptors may not only represent a pro-neuronal differentiation signal, but also induce proliferation and self-renewal of NSCs [211,212,236].

In pathological conditions, where neurogenesis has been found to be disrupted, such as epilepsy, Alzheimer’s, or Parkinson’s Diseases, disturbances associated with ECS homeostasis were also found [203,237,238,239,240,241] (reviewed in References [113,242,243,244]). Thus, it may be proposed that an interplay between the rise of symptomatology of these disorders may be originated from a combined early impairment in neural development or aberrant adult neurogenesis with deficiencies in the ECS. In fact, genetic studies have put in evidence that several genes are heavily implied in different brain disorders (like *HTT*, *SNCA*, or *PSEN1*), and which directly influence the modulation of neurogenesis [245,246,247], also modulate the ECS [248,249,250]. Therefore, changes associated with these genes will most probably impact the ontogeny of these disorders.

How cannabinoids modulate neurogenesis in physiological, epileptic, and other pathological conditions (in both in vitro and in vivo) is further reviewed in References [43,243,251,252].

### 4.5. Current Cannabinoid Therapies for Epilepsy

Although there are several pieces of evidence showing that the activation of CB1R can ameliorate seizure outcome, due to its psychotomimetic effects, its use has been limited. Therefore, CB2R and other non-psychotomimetic receptors, such as TRPV1, may prove an alternative efficient target. In fact, epidemiological data and case reports have increasingly depicted the overall positive effects of cannabinoid administration using a high ratio of CBD:THC in the management of pharmacoresistant epilepsy (reviewed in References [10,253,254]). In an elegant review by Rosenberg and colleagues, there are currently 181 animal models of epilepsy, 111 acute models of seizures and epilepsy, and 70 chronic models of epileptogenesis, which have been subject to cannabinoid pre-clinical testing and research [255]. These have been divided according to their effects as modulators of the ECS, CB1R/CB2R agonists and antagonists, the action of Δ^9^-THC and CBD/CBDV.

eCBs can be increased by inhibiting degrading enzymes. In fact, inhibition of FAAH by the synthetic blocker URB597, which promotes an increase in the endogenous levels of AEA, can result in reduced seizure severity, duration, and amplitude in animal models of epilepsy, as well as protecting hippocampal neurons against damage [184,256,257,258]. However, these effects have been shown to be AEA-dose dependent. When AEA is exogenously administered to PTZ-induced seizure mice, higher doses have been shown to be pro-convulsant and low doses anti-convulsant. These contrasting responses were revealed as being TRPV1-dependent, as at higher doses, AEA is able to activate this receptor [259]. In the presence of capsazepine (TRPV1 antagonist), AEA had anti-convulsant effects even at high doses, whilst in the presence of AM251 (CB1R antagonist), AEA potentiated TRPV1-mediated responses, namely, decreasing the latencies to the onset of PTZ-induced seizures [259]. Contrastingly, in the mouse MES and PTZ models, the MAGL inhibitor SAR127303, which promotes an increase in the endogenous levels of 2-AG, produced no protective activity against seizures. Nonetheless, repeated exposure to SAR127303 in the kindling mouse model slowed down epileptogenesis, as seizure severity proved to be significantly lower as compared to control animals [260]. However, in the PTZ mouse model, seizure incidence and severity decreased by increasing 2-AG levels by blocking the 2-AG degrading enzyme ABHD6 with WWL123 [261].

**Figure 5:**
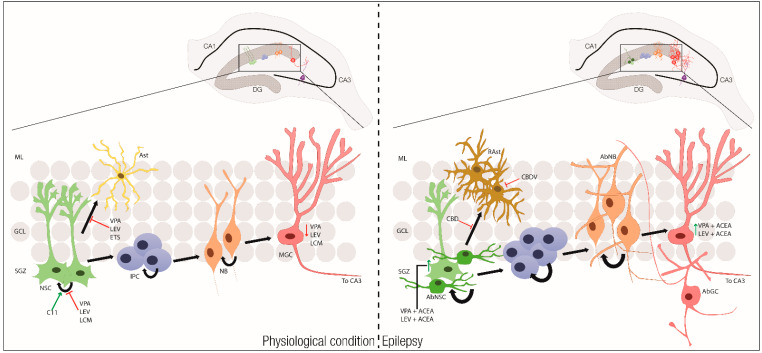
The effect of antiepileptic drugs (AEDs) and cannabinoids in physiological conditions and epilepsy. In physiological conditions (left), SGZ NSCs divide and ultimately differentiate into mature granule cells (MGC) that are able to make functional connections with the CA3 region of the hippocampus. NSCs can also generate astrocytes (Ast). AEDs, such as VPA, LEV, and LCM, decrease NSC proliferation and diminishes the maturation of MGC. Differentiation into astrocytes is blocked by VPA, LEV, and ETS. In epilepsy (right), NSCs fails to divide correctly and generate abnormal NSCs (AbNSC). On the other way, SGZ NSCs can ultimately convert into reactive astrocytes (Rast). The uncontrolled and excessive production of IPCs will translate into defective aberrant neuroblasts (AbNB) and new aberrant neurons (AbGC) with altered morphological and electrophysiological properties. The proliferative capacity of normal NSCs is compromised, leading to a depletion of the NSC population, resulting in diminished neurogenesis overtime. ACEA, when combined with VPA and LEV, restores to normal levels, NSC proliferation and promotes maturation of MGC. CBD inhibits proliferation and differentiation of Rasts, while CBDV decreases their reactivity. ACEA = arachidonyl-2′-chloroethylamide; C11 = hybrid antiepileptic compound; CBD = cannabidiol; CBDV = cannabidivarin; ETS = ethosuximide; IPC = Intermediate progenitor cells; LCM = lacosamide; LEV = levetiracetam; MGC = Mature Granule cell; ML = Molecular layer; NB = Neuroblast; NSCs = Neural Stem Cells; SGZ = Subgranular Zone; VPA = valproic acid.

As mentioned in previous chapters, neurogenesis is compromised in epilepsy. Combined therapies with cannabinoids and AEDs have been tested to assess the potential benefits of a conjoint treatment. Arachidonyl-2′-chloroethylamide (ACEA) has been tested with the AEDs valproic acid, levetiracetam, ethosuximide, lacosamide, or C11 (a new hybrid compound made from levetiracetam, ethosuximide, and lacosamide) in different models of induced-seizures, and were able to recover proliferation of NSCs and to promote neuronal differentiation [262,263,264,265,266,267] (Figure 5). Treatment with WIN 55,212-2 was shown to prevent chronic epileptic hippocampal damage in rat [268,269] and mouse [270,271] models by attenuating the severity and frequency of spontaneous recurrent seizures. This compound also increased the antiepileptic effects of AED, such as valproic acid, carbamazepine, phenobarbital, phenytoin, lamotrigine, pregabalin, and topiramate [272,273]. Similar results were obtained with the CB1R agonist ACEA [274], which also increased the seizure threshold [275]. Interestingly, the ACEA antiepileptic effects can be potentiated by nitric oxide (NO) precursor L-arginine and blocked by nitric oxide synthase (NOS) inhibitor L-NAME or selective neuronal NOS inhibitor 7-NI [276].

CB1R antagonists displayed pro-epileptic effects in pre-clinical seizure models [277]. Additionally to the pharmacological modulation of cannabinoid receptors, studies using conditional CB1R KO models have demonstrated that ECS signaling, depending on the neuronal subpopulation, plays an important role in the termination of epileptic activity, whereas having no impact in the initiation of hyperexcitability [189]. While most studies seem to point for antiepileptic properties of CB2R activation (reviewed in Reference [168]), there are studies that also suggest that CB2R may have pro-epileptic effects. One study showed that activation of CB2R alone with AM1241 agonist increased the severity and frequency of seizures in rats subjected to the PTZ paradigm [278]. Curiously, CB2R antagonist AM630 alone had no effect on PTZ induced-seizure rats [279].

Δ^9^-THC has a more complex mode of action than synthetic cannabinoids since it acts as a partial CB1R/CB2R agonist, and it also activates TRP channels. Indeed, data regarding Δ^9^-THC in epilepsy is contradictory, and its effects appear to be dose and model-dependent being either anti- and pro-epileptic [270,280,281,282]. Remarkably, administration of Δ^9^-THC on pregnant female mice during embryonic days 12.5 through 16.5, which are critical days for cortical neurogenesis and migration of neurons, reduced seizure latency in the offspring [283]. A similar experimental setting but performed in rats showed profound alterations in GABAergic and glutamatergic transmission, as well as disruptions in the uptake and expression of transporters of these neurotransmitters [284,285]. Disruptions in GABAergic and glutamatergic systems have already been extensively reported and implicated in epilepsy (reviewed in Reference [5]).

CBDV, a recent rediscovered non-psychotomimetic cannabinoid, has been capturing the interest of researchers, due to the variety of functions it can modulate. It has been shown to reduce seizure severity and mortality, to decrease neuronal loss and astrocyte reactivity, and to potentiate AEDs, such as valproic acid and ethosuximide [286,287,288].

CBD primarily displays antiepileptic effects [289]. These properties have been explained, due to the low affinity to CB1R/CB2Rs and to being able to activate other non-canonical cannabinoid receptors, such as GPR55 [178]. Besides its antiepileptic effects, CA3 cell death, a hallmark of epilepsy pathology, was shown to be significantly reduced after CBD administration [290]. Importantly, hippocampal interneuron functions can be restored, and the aberrant reactive astrocytes can be inhibited with CBD [291,292]. The anti-inflammatory effect of CBD has also been associated with potential synergistic crosstalk between cannabinoid receptors and adenosine type 1/2A receptors (A1R and A2AR) [293,294,295,296]. In fact, a crosstalk between the endocannabinoid and the adenosinergic systems has been hinted in epilepsy [297,298]. Indeed, the cannabinoid-adenosine-mediated anti-inflammatory properties have been described to involve A2AR-CB2R heteromers in microglia [299]. Crosstalk between microglia and neurons has also been suggested to regulate synaptic transmission, which is critical for the development of spontaneous seizures [300,301] (reviewed in References [302,303]).

Currently, there are four clinical trials involving cannabinoids and epilepsy, focusing mainly on the use of CBD as a therapeutic agent for pediatric epilepsy [304]. All of them agree that CBD is an effective alternative drug with no apparent side-effects, when combined with conventional AEDs, in pharmacoresistant patients. However, this needs to be further supported with long-term double-blind, randomized placebo-controlled trials [305]. Furthermore, because it interacts pharmacokinetically with a wide range of other therapeutic substances, the precise mechanism of action of CBD remains unclear and needs to be more deeply investigated.

## 5. Cannabinoids, Neural Stem Cells and Epilepsy: Perspectives and Concluding Remarks

In the present manuscript, we have reviewed how the ECS and AHN have their physiological functions jeopardized during epileptogenesis. Compromised neurogenesis has been extensively reported and reviewed in both patients and animal models of epilepsy, in which aberrant neurogenesis is observed and where the premature loss of NSCs occurs [54,55,306].

Current available AEDs are not effective for all types of epilepsy, as some patients exhibit strong pharmacoresistance. These patients can benefit from cannabinoid-derived drugs to ameliorate from epileptic symptoms when all other conventional therapies (e.g., AEDs) have failed. In fact, most patients report a significant reduction (between 50–90% less) in the frequency of seizures when under a combined cannabinoid-AED therapy [307]. Cannabinoids have been used as a therapy to treat seizures since the early 1800s, and although they have been used for other pathologies for millennia, the development of cannabinoid-based drugs has been very slow. This is mainly due to the psychotomimetic effects associated with *Cannabis* consumption. However, with the growing existence of several plant-derived and synthetic cannabinoids that have little or no psychotomimetic properties, the ECS harbors an immense uncovered potential to be used as a therapeutic agent for several neurological disorders, especially when combined with stem cell therapy [10,216,218,308,309,310].

The current pre-clinical data fail to completely explain the precise mechanisms by which cannabinoids and ECS modulation interact with AEDs, as it is difficult establishing coherent clinical trials. None of the current clinical trials for epilepsy are presently assessing the potential role of modulation of neurogenesis via the ECS. Concomitantly, choosing the best cannabinoid-based compound with the safest clinical effects is difficult, due to the wide array of cannabinoid actions in the organism.

Cannabinoid-based stem cell therapies come as a “secondary effect” of co-administration of cannabinoids with AEDs, as there is evidence that cannabinoids not only potentiate AEDs, but seem to inhibit the negative impact on neurogenesis caused by them. There are nonetheless some key aspects that need further studies, mainly whether the aberrant neurogenesis found in epilepsy is a consequence of the disease as a compensatory mechanism or is actually pathological and exacerbates the symptoms [311]. Notwithstanding, the promising results mentioned in this review suggest that NSC modulation by cannabinoids can be a potential therapeutic target in epilepsy.

The impact of cannabinoids on society is pushing more and more countries to legalize the use of cannabinoids for medicinal purposes, encouraging drug developers and the pharma industry to do more pre-clinical and clinical studies in the future. Certainly, combining both cannabinoid and NSC therapy will bring new knowledge in this field over the upcoming years, yielding a translational power with the potential to be impactful in both clinic and society. Therefore, innovative ideas that can place cannabinoids as one of the leading pharmacotherapeutic options to treat brain pathologies is imperative.

## Figures and Tables

**Table 1:** Neural Stem Cell therapies for epilepsy. Modulating miRNA, grafting human-induced pluripotent stem cells (hiPSC)-derived medial ganglionic eminence (MGE) cells in the hippocampus, and specific delivery of extracellular vesicles derived from mesenchymal stem cells (MSC) are potential therapies to be used as a treatment for epilepsy.

Neural Stem Cell Therapies for Epilepsy		
Modulating miRNA	Grafting Medial Ganglionic Eminence (MGE) Cells	Extracellular Vesicles Delivering
▪ 🠥 miR-22: 🠣 Seizure severity ▪ Silencing miR-134: 🠣 Seizure severity 🠣 Neuronal death in CA3 🠣 Mossy fiber sprouting ▪ Inhibiting miR-135a: 🠣 Seizure severity Restores the age-associated reduction of adult neurogenesis	▪ hiPSC-derived MGE: 🠣 Seizure severity 🠣 Mossy fiber sprouting 🠣 Ectopic hilar granule cells Preserved reelin^+^ neurons and interneurons 🠥 New-born cells 🠣 Cognitive impairments 🠣 Mood dysfunction	▪ EVs: 🠣 Loss of neurons and interneurons in DG and CA1 🠣 Number of ectopic new-born neurons Prevented cognitive impairment and pattern separation

## Supplementary Materials

The following are available online at https://www.mdpi.com/1422-0067/21/19/7309/s1, Table S1: Expression of cannabinoid receptors in the CNS and peripherical tissues. n.d = no data.
