# Supplementary material for: Neural Stem Cells and Cannabinoids in the Spotlight as Potential Therapy for Epilepsy
Source: Int J Mol Sci. 2020 Oct 3;21(19):7309. doi: 10.3390/ijms21197309 (PMC7582633; doi:10.3390/ijms21197309)
Supplement: Supplementary file 1 [file ijms-21-07309-s001.pdf]

Supplementary Table S1. Expression of cannabinoid receptors in the CNS and peripherical tissues. n.d = no data.

Location			CB1R	CB2R
Brain	Cortex	Layer I	[1,2]	n.d
		Layer II	[3,4]	n.d
		Layer III	[3,4]	[5]
		Layer IV	n.d	n.d
		Layer V	[3]	[5]
		Layer VI	[1,2]	n.d
	Olfactory Bulb		[6,7]	[8]
	Globus Pallidus		[9]	[5]
	Entopeduncular nucleus		[9]	n.d
	Striatum		[7]	[10]
	Substantia Nigra		[6,7,9]	[11]
	Hippocampus	CA1	[6,7]	[5,12,13]
		CA2	[6,7]	[12]
		CA3	[6,7]	[12,13]
		Dentate Gyrus	[6]	[12,13]
	Brainstem		[9]	[10,14]
	Entorhinal cortex		[7]	[10]
	Hypothalamus		[7,9,15]	[15,16]
	Nucleus accumbens		[7,17]	[17,18]
	Amygdala		[6,7,9,17]	[10,17–19]
	Cerebellum		[6,7]	[18]
Periphery	Immune System	Spleen	[20]	[5,18,20]
		Leukocytes	Not observed	[18,21]
		Tonsils	[22,23]	[23]
		Thymus	Not observed	[20]
	Reproductive System	Testis	[24–27]	[18,25–27]
		Ovaries	[28]	[28]
	Digestive System	Stomach	[29]	n.d
		Gut	[29,30]	[18,30]
		Liver	[31]	[32]
		Pancreas	[16]	[16]
	Cardiovascular System	Heart	[33]	[33]
	Respiratory System	Lungs	[23]	[23]

References

1. Buckley, N.E.; Hansson, S.; Harta, G.; Mezey, É. Expression of the CB1 and CB2 receptor messenger RNAs during embryonic development in the rat. *Neuroscience* **1997**, *82*, 1131–1149, doi:10.1016/S0306-4522(97)00348-5.
2. Romero, J.; Garcia-Palomero, E.; Berrendero, F.; Garcia-Gil, L.; Hernandez, M.L.; Ramos, J.A.; Fernández-Ruiz, J.J. Atypical location of cannabinoid receptors in white matter areas during rat brain development. *Synapse (New York, N.Y.)* **1997**, *26*, 317–323, doi:10.1002/(SICI)1098-2396(199707)26:3<317::AID-SYN12>3.0.CO;2-S.
3. Yeh, M.L.; Selvam, R.; Levine, E.S. BDNF-induced endocannabinoid release modulates neocortical glutamatergic neurotransmission. *Synapse* **2017**, *71*, e21962, doi:10.1002/syn.21962.
4. Lee, T.T.-Y.; Filipski, S.B.; Hill, M.N.; McEwen, B.S. Morphological and behavioral evidence for impaired prefrontal cortical function in female CB1 receptor deficient mice. *Behavioural Brain Research* **2014**, *271*, 106–110, doi:10.1016/j.bbr.2014.05.064.
5. Lanciego, J.L.; Barroso-Chinea, P.; Rico, A.J.; Conte-Perales, L.; Callén, L.; Roda, E.; Gómez-Bautista, V.; López, I.P.; Lluis, C.; Labandeira-García, J.L.; et al. Expression of the mRNA coding the cannabinoid receptor 2 in the pallidal complex of Macaca fascicularis. *Journal of Psychopharmacology* **2011**, *25*, 97–104, doi:10.1177/0269881110367732.
6. Pettit, D.A.D.; Harrison, M.P.; Olson, J.M.; Spencer, R.F.; Cabral, G.A. Immunohistochemical localization of the neural cannabinoid receptor in rat brain. *Journal of Neuroscience Research* **1998**, *51*, 391–402, doi:10.1002/(SICI)1097-4547(19980201)51:3<391::AID-JNR12>3.0.CO;2-A.
7. Moldrich, G.; Wenger, T. Localization of the CB1 cannabinoid receptor in the rat brain. An immunohistochemical study☆. *Peptides* **2000**, *21*, 1735–1742, doi:10.1016/S0196-9781(00)00324-7.
8. Gong, J.-P.; Onaivi, E.S.; Ishiguro, H.; Liu, Q.-R.; Tagliaferro, P.A.; Brusco, A.; Uhl, G.R. Cannabinoid CB2 receptors: Immunohistochemical localization in rat brain. *Brain Research* **2006**, *1071*, 10–23, doi:10.1016/j.brainres.2005.11.035.
9. Pertwee, R.G. The diverse CB1 and CB2 receptor pharmacology of three plant cannabinoids: Δ9-tetrahydrocannabinol, cannabidiol and Δ9-tetrahydrocannabivarin. *British Journal of Pharmacology* **2008**, *153*, 199–215, doi:10.1038/sj.bjp.0707442.
10. Chen, D.; Gao, M.; Gao, F.; Su, Q.; Wu, J. Brain cannabinoid receptor 2: expression, function and modulation. *Acta Pharmacologica Sinica* **2017**, *38*, 312–316, doi:10.1038/aps.2016.149.
11. Brusco, A.; Tagliaferro, P.A.; Saez, T.; Onaivi, E.S. Ultrastructural Localization of Neuronal Brain CB2 Cannabinoid Receptors. *Annals of the New York Academy of Sciences* **2008**, *1139*, 450–457, doi:10.1196/annals.1432.037.
12. Stempel, A.V.; Stumpf, A.; Zhang, H.-Y.; Özdoğan, T.; Pannasch, U.; Theis, A.-K.; Otte, D.-M.; Wojtalla, A.; Rácz, I.; Ponomarenko, A.; et al. Cannabinoid Type 2 Receptors Mediate a Cell Type-Specific Plasticity in the Hippocampus. *Neuron* **2016**, *90*, 795–809, doi:10.1016/j.neuron.2016.03.034.
13. Li, Y.; Kim, J. Neuronal expression of CB2 cannabinoid receptor mRNAs in the mouse hippocampus. *Neuroscience* **2015**, *311*, 253–267, doi:10.1016/j.neuroscience.2015.10.041.

14. Van Sickle, M.D. Identification and Functional Characterization of Brainstem Cannabinoid CB2 Receptors. *Science* **2005**, *310*, 329–332, doi:10.1126/science.1115740.
15. Xing, G.; Carlton, J.; Jiang, X.; Wen, J.; Jia, M.; Li, H. Differential Expression of Brain Cannabinoid Receptors between Repeatedly Stressed Males and Females may Play a Role in Age and Gender-Related Difference in Traumatic Brain Injury: Implications from Animal Studies. *Frontiers in Neurology* **2014**, *5*, doi:10.3389/fneur.2014.00161.
16. Romero-Zerbo, S.Y.; Garcia-Gutierrez, M.S.; Suárez, J.; Rivera, P.; Ruz-Maldonado, I.; Vida, M.; Rodriguez de Fonseca, F.; Manzanares, J.; Bermúdez-Silva, F.J. Overexpression of Cannabinoid CB2 Receptor in the Brain Induces Hyperglycaemia and a Lean Phenotype in Adult Mice: Brain CB2 overexpression, hyperglycaemia and thinness. *Journal of Neuroendocrinology* **2012**, *24*, 1106–1119, doi:10.1111/j.1365-2826.2012.02325.x.
17. Navarrete, F.; Pérez-Ortiz, J.M.; Manzanares, J. Cannabinoid CB2 receptor-mediated regulation of impulsive-like behaviour in DBA/2 mice: CB2 receptor and impulsivity. *British Journal of Pharmacology* **2012**, *165*, 260–273, doi:10.1111/j.1476-5381.2011.01542.x.
18. Liu, Q.-R.; Pan, C.-H.; Hishimoto, A.; Li, C.-Y.; Xi, Z.-X.; Llorente-Berzal, A.; Viveros, M.-P.; Ishiguro, H.; Arinami, T.; Onaivi, E.S.; et al. Species differences in cannabinoid receptor 2 (CNR2 gene): identification of novel human and rodent CB2 isoforms, differential tissue expression and regulation by cannabinoid receptor ligands. *Genes, Brain and Behavior* **2009**, *8*, 519–530, doi:10.1111/j.1601-183X.2009.00498.x.
19. García-Gutiérrez, M.S.; García-Bueno, B.; Zoppi, S.; Leza, J.C.; Manzanares, J. Chronic blockade of cannabinoid CB2 receptors induces anxiolytic-like actions associated with alterations in GABAA receptors: Chronic blockade of CB2 receptors is anxiolytic. *British Journal of Pharmacology* **2012**, *165*, 951–964, doi:10.1111/j.1476-5381.2011.01625.x.
20. Schatz, A.R.; Lee, M.; Condie, R.B.; Pulaski, J.T.; Kaminski, N.E. Cannabinoid Receptors CB1 and CB2: A Characterization of Expression and Adenylate Cyclase Modulation within the Immune System. *Toxicology and Applied Pharmacology* **1997**, *142*, 278–287, doi:10.1006/taap.1996.8034.
21. Castaneda, J.T.; Harui, A.; Roth, M.D. Regulation of Cell Surface CB2 Receptor during Human B Cell Activation and Differentiation. *Journal of Neuroimmune Pharmacology* **2017**, *12*, 544–554, doi:10.1007/s11481-017-9744-7.
22. Martín-Fontecha, M.; Angelina, A.; Rückert, B.; Rueda-Zubiaurre, A.; Martín-Cruz, L.; van de Veen, W.; Akdis, M.; Ortega-Gutiérrez, S.; López-Rodríguez, M.L.; Akdis, C.A.; et al. A Fluorescent Probe to Unravel Functional Features of Cannabinoid Receptor CB1 in Human Blood and Tonsil Immune System Cells. *Bioconjugate Chemistry* **2018**, *29*, 382–389, doi:10.1021/acs.bioconjchem.7b00680.
23. Galiegue, S.; Mary, S.; Marchand, J.; Dussossoy, D.; Carriere, D.; Carayon, P.; Bouaboula, M.; Shire, D.; Fur, G.; Casellas, P. Expression of Central and Peripheral Cannabinoid Receptors in Human Immune Tissues and Leukocyte Subpopulations. *European Journal of Biochemistry* **1995**, *232*, 54–61, doi:10.1111/j.1432-1033.1995.tb20780.x.
24. Meccariello, R.; Battista, N.; Bradshaw, H.B.; Wang, H. Updates in Reproduction Coming from the Endocannabinoid System. *International Journal of Endocrinology* **2014**, *2014*, 1–16, doi:10.1155/2014/412354.

25. Bovolin, P.; Cottone, E.; Pomatto, V.; Fasano, S.; Pierantoni, R.; Cobellis, G.; Meccariello, R. Endocannabinoids are Involved in Male Vertebrate Reproduction: Regulatory Mechanisms at Central and Gonadal Level. *Frontiers in Endocrinology* **2014**, *5*, doi:10.3389/fendo.2014.00054.
26. Nielsen, J.E.; Rolland, A.D.; Rajpert-De Meyts, E.; Janfelt, C.; Jørgensen, A.; Winge, S.B.; Kristensen, D.M.; Juul, A.; Chalmel, F.; Jégou, B.; et al. Characterisation and localisation of the endocannabinoid system components in the adult human testis. *Scientific Reports* **2019**, *9*, doi:10.1038/s41598-019-49177-y.
27. Grimaldi, P.; Orlando, P.; Di Siena, S.; Lolicato, F.; Petrosino, S.; Bisogno, T.; Geremia, R.; De Petrocellis, L.; Di Marzo, V. The endocannabinoid system and pivotal role of the CB2 receptor in mouse spermatogenesis. *Proceedings of the National Academy of Sciences* **2009**, *106*, 11131–11136, doi:10.1073/pnas.0812789106.
28. El-Talatini, M.R.; Taylor, A.H.; Elson, J.C.; Brown, L.; Davidson, A.C.; Konje, J.C. Localisation and Function of the Endocannabinoid System in the Human Ovary. *PLoS ONE* **2009**, *4*, e4579, doi:10.1371/journal.pone.0004579.
29. Hornby, P.J.; Prouty, S.M. Involvement of cannabinoid receptors in gut motility and visceral perception. *British Journal of Pharmacology* **2004**, *141*, 1335–1345, doi:10.1038/sj.bjp.0705783.
30. Battista, N.; Di Sabatino, A.; Di Tommaso, M.; Biancheri, P.; Rapino, C.; Giuffrida, P.; Papadia, C.; Montana, C.; Pasini, A.; Vanoli, A.; et al. Altered Expression of Type-1 and Type-2 Cannabinoid Receptors in Celiac Disease. *PLoS ONE* **2013**, *8*, e62078, doi:10.1371/journal.pone.0062078.
31. Mukhopadhyay, B.; Liu, J.; Osei-Hyiaman, D.; Godlewski, G.; Mukhopadhyay, P.; Wang, L.; Jeong, W.-I.; Gao, B.; Duester, G.; Mackie, K.; et al. Transcriptional Regulation of Cannabinoid Receptor-1 Expression in the Liver by Retinoic Acid Acting via Retinoic Acid Receptor-γ. *Journal of Biological Chemistry* **2010**, *285*, 19002–19011, doi:10.1074/jbc.M109.068460.
32. Buckley, N.E. The peripheral cannabinoid receptor knockout mice: an update: The CB2R knockout mice. *British Journal of Pharmacology* **2008**, *153*, 309–318, doi:10.1038/sj.bjp.0707527.
33. Kaschina, E. Cannabinoid CB1/CB2 Receptors in the Heart: Expression, Regulation, and Function. In *Cannabinoids in Health and Disease*; Meccariello, R., Chianese, R., Eds.; InTech, 2016 ISBN 978-953-51-2429-0.
